# UPLC-QTOF-MS with chemical profiling approach for rapidly evaluating chemical consistency between traditional and dispensing granule decoctions of Tao-Hong-Si-Wu decoction

**DOI:** 10.1186/1752-153X-6-143

**Published:** 2012-11-24

**Authors:** Erxin Shang, Zhenhua Zhu, Li Liu, Yuping Tang, Jin-ao Duan

**Affiliations:** 1 Jiangsu Key Laboratory for High Technology Research of TCM Formulae, Nanjing University of Chinese Medicine, Nanjing, 210046, China; 2 School of Pharmacy, Guangdong Medical College, Dongguan, 523808, China

**Keywords:** UPLC-QTOF-MS, Tao-Hong-Si-Wu decoction, Dispensing granule

## Abstract

**Background:**

In the present study, chemical consistency between traditional and dispensing granule decoctions of Tao-Hong-Si-Wu decoction was rapidly evaluated by UPLC-QTOF-MS coupled with the MarkerLynx software. Two different kinds of decoctions, namely traditional decoction: water extract of mixed six constituent herbs of Tao-Hong-Si-Wu decoction, and dispensing granules decoction: mixed water extract of each individual herbs of Tao-Hong-Si-Wu decoction, were prepared.

**Results:**

Chemical difference was found between traditional and dispensing granule decoctions, and albiflorin, paeoniflorin, gallic acid, amygdalin, and hydroxysafflor yellow A were identified as the significantly changed components during decocting Tao-Hong-Si-Wu decoction. All the peaks of mass spectrum from Tao-Hong-Si-Wu decoction and each herb were extracted and integration by using QuanLynx™. And the optimized data was used for linear regression analysis. The contribution of each herb in Tao-Hong-Si-Wu decoction, and the optimal compatibility proportion of dispensing granule decoction were derived from the linear regression equation.

**Conclusions:**

The optimal dosage proportionality of Tao-Hong-Si-Wu dispensing granule decoction was obtained as 2.5:0.2:1:0.5:0.6:0.1 (DG : CX : BS : SD : TR : HH), which guided better clinic application of Tao-Hong-Si-Wu decoction as dispensing granule decoctions usage, and it also provided some experimental data to reveal the compatibility rule of the relative TCM formulae.

## Background

Traditional Chinese medicine (TCM) originated in ancient China and has evolved over thousands of years. TCM decoction is the earliest and most widely used form. Different kinds of herbs are mixed together and boiled with water to get decoction, which are usually took by patients themselves. There are some advantages for using decoction, such as effective, absorbed quickly and completely with high bioavailability. However, the decoction is trouble to boil, inconvenient to carry, store and take, which inhibit its clinic application [1].

The granule of individual herb is called dispensing granule. With the improvement of scientific and technological level, dispensing granules decoction is developed as an alternative to decoctions, prescribed by traditional medicine practitioners in Japan, Korea, Singapore, Hong Kong, Taiwan and Mainland China, or even in the United States and some European countries. Taking Chinese herbal medicine as raw materials, dispensing granules decoction was mainly prepared through extraction, concentration, drying and granulation procedures [2]. It not only has the advantages of the traditional Chinese medicine decoction, but also is convenient to carry, store, and take for patients [3]. Patients could directly mix and dissolve different dispensing granules with hot water in accordance with the recipe of combinatorial formulae to get dispensing granules decoction. However, a debate has continued since the emergence of dispensing granules. The focus of the debate is that the chemical components of dispensing granules decoction may be different from those of traditional decoction, which make their efficacy not equivalent. In fact, a few cases on content changes in marker compounds or chemical consistency during boiling of combinatorial formula have been reported [4-7], which revealed there were differences between dispensing granule decoction and traditional decoction, but no mention of further solution methods.

Modern chromatographic methods such as high performance liquid chromatography (HPLC) using a few compounds as markers have been developed to qualitatively or quantitatively compare the quality of dispensing granule decoction and traditional Chinese medicine decoction [4,5,8]. Song-Lin Li [7] proposed and validated an ultra performance liquid chromatography coupled with photo-diode array detector and time-of-flight mass spectrometry (UPLC-PDA-TOF-MS) based chemical profiling approach to rapidly evaluate chemical consistency between traditional and dispensing granule decoctions of traditional medicine combinatorial formulae using San-Huang-Xie-Xin-Tang (SHXXT) as a model combinatorial formula. In recent years, UPLC-QTOF-MS has been increasingly used for study of many herbs [9-11].

Tao-Hong-Si-Wu decoction is a commonly used traditional combinatorial formula composed of Angelicae Sinensis Radix (Danggui, DG), Chuanxiong Rhizoma (Chuanxiong, CX), Paeoniae Radix Alba (Baishao, BS), Rehmanniae Radix Praeparata (Shudi, SD), Persicae Semen (Taoren, TR) and Carthami Flos (Honghua, HH), which is widely applied in clinical practice for treating primary dysmenorrhea [12]. Tao-Hong-Si-Wu decoction contains many bio-active constituents, such as ferulic acid, senkyunolide I, paeoniflorin, amygdalin, hydroxysafflor yellow A and so on. Ferulic acid, a characteristic aromatic acid in both DG and CH, has been reported that it could significantly improve blood fluidity, inhibit platelet aggregation, decrease serum lipids, prevent thrombus formation, protect neuron like PC12 cells, and exhibit strong antioxidant activity [13-18]. Senkyunolide I, a phthalide in both DG and CX, has been exhibited the activity of reducing the metamorphose damage of the red blood cell caused by ConA [19]. Paeoniflorin, a monoterpene glycoside in BS has been showed many pharmacological effects, such as anticancer, anti-proliferative and neuroprotective [20,21]. Amygdalin, a quality marker of TR, has been reported to treat asthma, aplastic anemia and tumors in oriental medicine [22]. Hydroxysafflor yellow A, a quality marker of HH, has been demonstrated the activities of antioxidation, myocardial and cerebral protective effects [23-25]. Hydroxysafflor yellow A has also been demonstrated with a strong antagonistic effect on platelet activating factor receptor [26].

In this paper, chemical consistency between traditional and dispensing granule decoctions of Tao-Hong-Si-Wu decoction was rapidly evaluated by UPLC-QTOF-MS coupled with the MarkerLynx software. Under the chromatographic and MS conditions, the significantly changed components were identified or tentatively assigned by comparing their mass spectrums with the LC-MS/MS library. All the peaks of mass spectrum from Tao-Hong-Si-Wu decoction and each herb were extracted and integration by using QuanLynx™. And the optimized data was used for linear regression analysis. The contribution of each herb in Tao-Hong-Si-Wu decoction, and the optimal compatibility proportion of dispensing granule decoction were derived from the linear regression equation.

## Experimental

### Chemicals, solvents and herbal materials

HPLC-grade acetonitrile and formic acid were purchased from Merck (Merck, Darmstadt, Germany); ultra-pure water was purified by an EPED super purification system (EPED, Nanjing, China). The distilled water was used for the extraction and preparation of samples.

DG was collected in July 2011 from Min Xian (Gansu, China). CX, BS, SD, TR and HH were all purchased from Nanjing Medicinal Material Company (Nanjing, China). The crude plant of All herbs were identified as *Angelica sinensis* (Oliv.) Diels, *Ligusticum chuanxiong* Hort., *Paeonia lactiflora* Pall., *Rehmannia glutinosa* Libosch., *Prunus persica* (L.) Batsch and *Carthamus tinctorius* L. by the corresponding author. The voucher specimens (No. NJUTCM-20110916-20110921) were deposited in the Herbarium of Nanjing University of Chinese Medicine, China. These herbs were the material sources for preparing traditional and dispensing granule decoctions of Tao-Hong-Si-Wu decoction in the study.

### Liquid chromatography

UPLC was performed on a Waters ACQUITY UPLC™ system (Waters, Milford, MA, USA), equipped with a binary solvent delivery system, an auto-sampler, and a photodiode-array detection (PDA) system. The chromatography was performed by using ACQUITY BEH C_18_ (100 mm × 2.1 mm, 1.7 μm, Waters, Milford, MA, USA) column. The mobile phase consisted of (A) water containing 0.1% formic acid and (B) acetonitrile. The UPLC eluting conditions were optimized as follows: linear gradient elution from 1% to 4% B (0–3.5 min), 4% to 4% B (3.5–9 min), 4% to 7% B (9–9.2 min), 7% to 17% B (9.2–15 min), 17% to 17% B (15–24 min), 17% to 30% B (24–27 min), 30% to 40% B (27–33 min), 40% to 50% B (33–36 min), 50% to 100% B (36–39 min), isocratic 100% B for 2 min, and then back to 1% B within 1 min. The flow rate was 0.4 ml·min^−1^. The temperature of column and auto-sampler maintained at 35°C and 10°C, respectively. Each wash cycle consisted of 200 μl strong solvent (85% ACN) and 600 μl weak solvent (10% ACN). The injection volume was 2 μl. The scan range for PDA was 190–400 nm.

### Mass spectrometry

Mass spectrometry was performed on a Waters SYNAPT™ Mass Spectrometry equipped with an electrospray ionization (ESI) source. The nebulization gas was set at 600 l·h^−1^. At temperature of 350°C, the cone gas was set at 50 l·h^−1^, and the source temperature was set at 120°C. Detection was performed in both positive and negative ion modes in the *m*/*z* range of 100–1000 Da, with an acquisition time of 0.5 s in centroid mode. The ESI conditions were as follows: capillary voltage 3000 V, cone voltage 30 V, source temperature 120°C, desolvation temperature 350°C, cone gas flow 50 l·h^−1^, and desolvation gas flow 600 l·h^−1^.

### Accurate mass measurement

All MS data were acquired by using the LockSpray™ to ensure mass accuracy and reproducibility. The [M − H]^−^and [M + H]^+^ ions of Leucine-enkephalin at m/z 554.2615 and m/z 556.2771 were used as the lock mass in negative and positive electrospray ionization modes, respectively. The concentration of Leucine-enkephalin was 2 μg·ml^−1^ and the infusion flow rate was 0.4 ml·min^−1^. Centroided data were acquired for each sample from 100 to 1000 Da, and dynamic range enhancement (DRE™) was applied throughout the MS experiment to ensure accurate mass measurement over a wide dynamic range. The data were processed by using MassLynx™ 4.1 software.

### Sample preparation

For traditional decoction, the 102 g of mixed crude herbs DG, CX, BS, SD, TR and HH at the weight ratio of 3:3:3:3:3:2 were crushed into small pieces, and then extracted twice in 1 and 0.8 L of water, with refluxing times of 2 and 1.5 h, respectively. The decoction was combined and the solvent was removed below 65°C till certain volume at the ratio of 1:1 (w/w, weight of all herbs and the extracted filtrates) under vacuum, and then ethanol was added slowly with churning all the time until the ethanol content reached 80%. After being deposited for 24 h, the solution was filtered to dispose of the deposition and concentrated to a certain concentration under vacuum below 65°C, and the sample of THSWD was obtained (1 g sample was equivalent to 3.17 g crude drugs of the formula). For dispensing granule decoction, the samples were prepared in our laboratory following common producing procedure. Water extraction of each individual herb (DG, CX, BS, SD, TR and HH) was the same as THSWD (1.0 g sample was equivalent to 2.28, 2.73, 5.21, 2.25, 8.40 and 2.67 g crude drugs of them, respectively). After sample concentration the dispensing granule came into being from water extracts by freezing drying. THSWDH of dispensing granule decoction was prepared by mixing the dispensing granule of each individual herb at the weight ratio of THSWD and the dispensing granules were resolved and filtered.

### Peak assignment

Peak assignment was performed by comparison with the data of HPLC-MS library for traditional Chinese medicine (TCM) formulae ingredients which was established on QTOF-MS with ESI interface by Jiangsu Key Laboratory for Traditional Chinese Medicine Formulae Research [12].

### Multivariate statistical analysis

The UPLC-TOF-MS data of all determined samples were analyzed by MarkerLynx software (Waters, Manchester, UK) to reveal any possible interacted components in decoctions of traditional medicine combinatorial formulae. For data collection, the parameters were set as follows: retention time ranging from 0 to 43 min, mass ranging from 100 to 1000 Da, mass tolerance at 0.02 Da. For peak integration, peak width at 5% of the height was 1 s, peak-to-peak baseline noise was 0, and peak intensity threshold was 10. No specific mass or adduct was excluded. For data analysis, a list of the intensities of the detected peaks was generated by using retention time (t_R_) and mass data (m/z) pairs as the identifier of each peak. An arbitrary ID was assigned to each t_R_-m/z pair in the order of their UPLC elution for data alignment. This was repeated for each run until the final sample. The data for the entire samples was sorted such that, for each sample, the correct peak intensity data for each t_R_-m/z pair was aligned in a table. The ion intensities for each detected peak were normalized against the sum of the peak intensities within that sample by using MarkerLynx. Ions of different samples were considered to be the same ion when they demonstrated the same t_R_ (tolerance of 0.01 min) and m/z value (tolerance of 0.02 Da). If a peak was not detected in a sample, the ion intensity was documented as zero in the table. The resulting three-dimensional data consisted of peak number (t_R_–m/z pair), sample name and ion intensity were analyzed by supervised orthogonal partial least squared discriminant analysis (OPLS–DA) by using the MarkerLynx software.

## Results and discussion

### Chromatographic conditions and TOFMS method development

**Figure 1:**
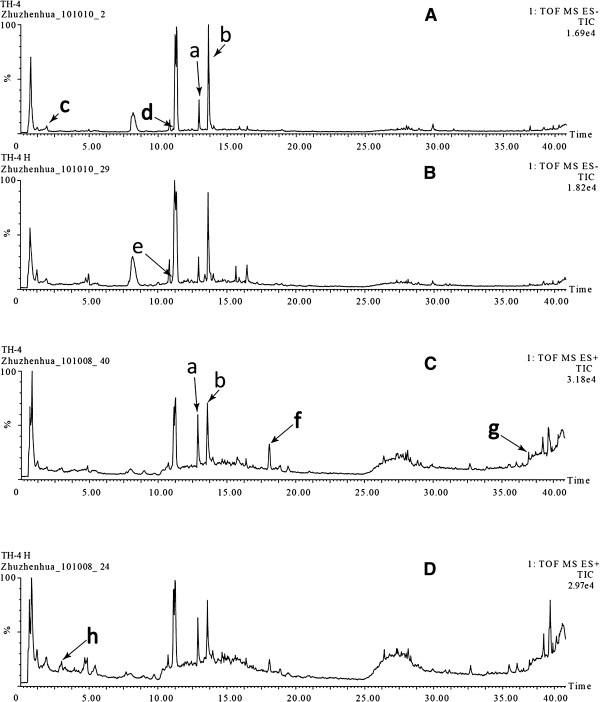
**Representative chromatograms of Tao-Hong-Si-Wu decoction.A**: chromatogram of traditional decoction (monitored in negative ion mode); **B**: chromatogram of dispensing granule decoction (monitored in negative ion mode); **C**: chromatogram of traditional decoction (monitored in positive ion mode); **D**: chromatogram of dispensing granule decoction (monitored in positive ion mode). The identified components (**a**-**h**) were marked in these chromatograms.

In the present study, different kinds of mobile phases, such as organic phase (Acetonitrile, Methanol) with a variety of aqueous phase (water, water containing formic acid, water containing triethylamine, water containing formic acid and ammonium) were tested. It was found that the mixture of acetonitrile-water (containing 0.1% formic acid) was a suitable mobile phase, which not only had good peak pattern, resolution, response value, but also could simultaneously separate major components in THSWD. The gradient elution profile and MS conditions were optimized with respect to the separation of major peaks and the sensitivity of MS detector. Under the optimized chromatographic and MS conditions, the major components in THSWD and THSWDH were well separated and detected within 43 min. The representative chromatograms monitored by MS were shown in Figure 1.

### Multivariate statistical analysis and chemical consistency evaluation

**Figure 2:**
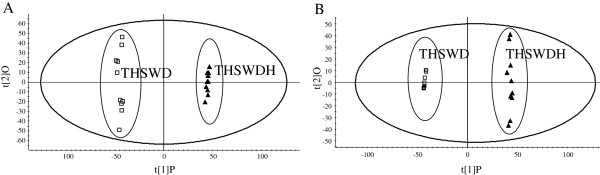
**OPLS–DA/Scores plot of THSWD and THSWDH obtained using Pareto scaling with mean centering.** (**A**) Negative ion mode; (**B**) positive ion mode; (square)THSWD; (*black triangle*)THSWDH.

To compare the chemical composition in THSWD and THSWDH, supervised orthogonal partial least squared discriminant analysis (OPLS–DA) was performed. After Pareto scaling with mean-centering, the data from both positive and negative ion modes were displayed as scores plots (Figure 2). The scores plots showed that the determined samples were clearly clustered into two groups, i.e. the THSWD and the THSWDH, indicating that the different processing procedures caused changes between traditional and dispensing granule decoctions of Tao-Hong-Si-Wu decoction.

**Figure 3:**
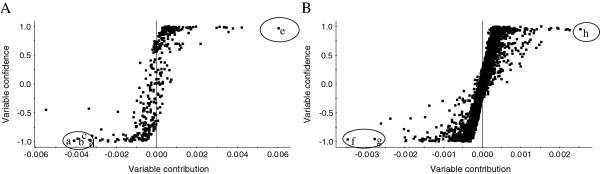
**OPLS–DA/S-Plot of THSWD and THSWDH obtained using Pareto scaling with mean centering.** (**A**) negative ion mode; (**B**) positive ion mode. **a** (t_*R*_ 12.97 min, *m*/*z* 525.1514), **b** (t_*R*_ 13.65 min, *m*/*z* 525.1459), **c** (t_*R*_ 13.64 min, *m*/*z* 449.1305), **d** (t_*R*_ 11.17 min, *m*/*z* 502.1556), **e** (t_*R*_ 11.20 min, *m*/*z* 611.1490), **f** (t_*R*_ 18.08 min, *m*/*z* 207.1004), **g** (t_*R*_ 36.97 min, *m*/*z* 191.1061) and **h** (t_*R*_ 3.02 min, *m*/*z* 120.0809).

To find out components contributing to the significant difference between THSWD and THSWDH, the extending statistical analysis was performed to generate S-plot (Figure 3). In the S-plot, each point represents an ion t_R_–*m*/*z* pair; the X-axis represents variable contribution, when the distance of the ion t_R_–*m*/*z* pair points is farther from zero, the ion has more contribution to the difference between the two groups; the Y-axis represents variable confidence, when the distance of the ion t_R_–*m*/*z* pair points is farther from zero, the ion has higher confidence level for the difference between two groups. Thus, the t_R_–*m*/*z* pair points at the two ends of “S” represent characteristic markers with the most confidence to each group.

According to the S-plot, six ions (**a**-**d**, **f**-**g**) at the bottom left corner of “S” were the ions contributing most to difference between THSWD and THSWDH. It was found that ions **a** (t_*R*_ 12.97 min,*m*/*z* 525.1514), **b** (t_*R*_ 13.65 min,*m*/*z* 525.1459), **c** (t_*R*_ 13.64 min,*m*/*z* 449.1305), **d** (t_*R*_ 11.17 min, *m*/*z* 502.1556), **e** (t_*R*_ 11.20 min, *m*/*z* 611.1490), **f** (t_*R*_ 18.08 min, *m*/*z* 207.1004), and **g** (t_*R*_ 36.97 min, *m*/*z* 191.1061) were detected with higher intensity in THSWD than in THSWDH, which indicated that the content of components correlating to ion **a**–**d** and **f**–**g** were lower in THSWDH than in THSWD.

Similarly, two ions e (t_*R*_ 11.20 min, *m*/*z* 611.1490) and h (t_*R*_ 3.02 min, *m*/*z* 120.0809) at the top right corner of “S” were the ions contributing most to the difference between THSWD and THSWDH. The intensity trends showed that the two ions could be detected with higher intensity in THSWDH than in THSWD, which suggested that the contents of compounds e and h were higher in THSWDH than in THSWD. All of the above results demonstrated that the contents of at least seven components were obviously different from traditional and dispensing granule decoctions of Tao-Hong-Si-Wu decoction.

### Identity assignment and confirmation of the significantly changed components

**Figure 4:**
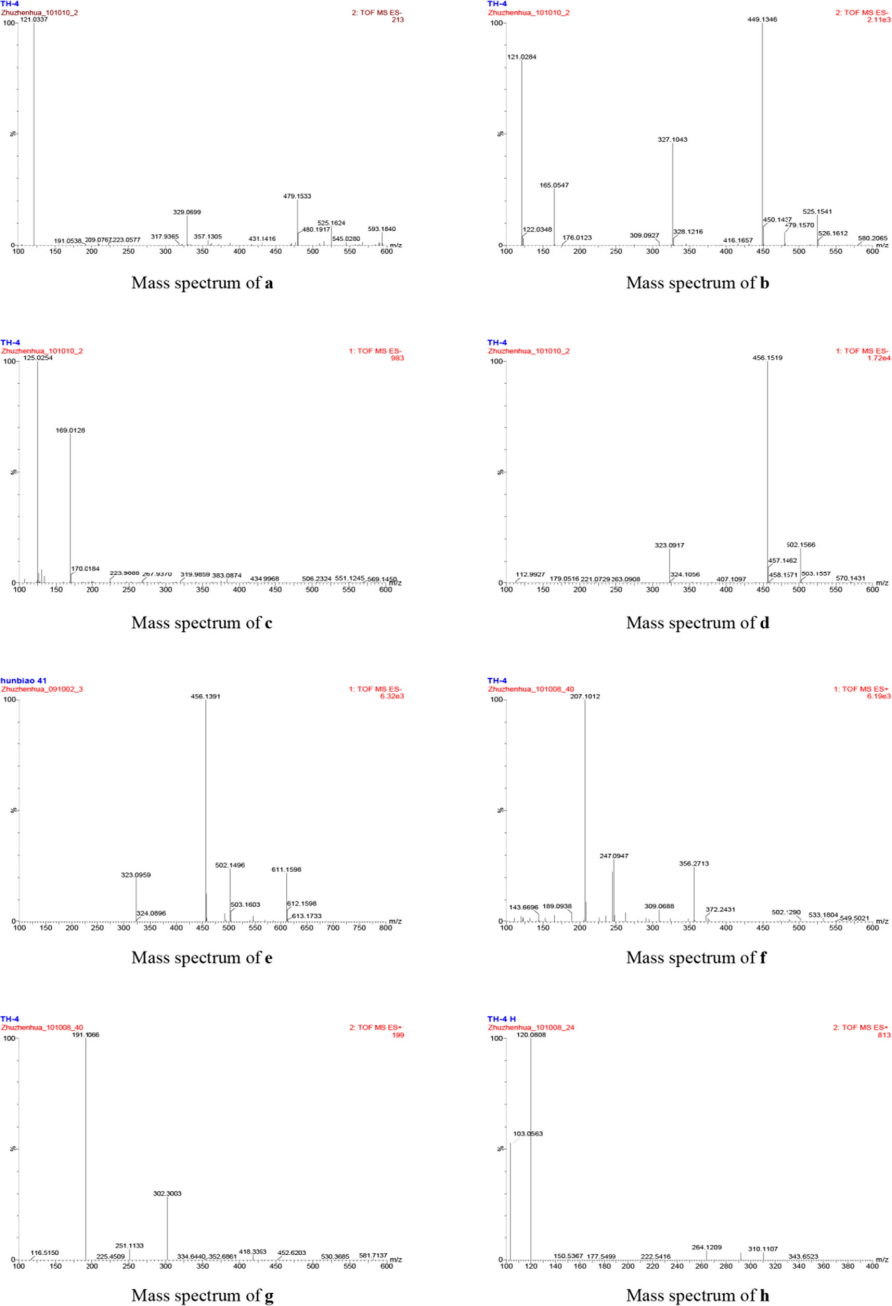
**Mass spectra of the significantly changed components (a-h) between traditional and dispensing granule Tao-Hong-Si-Wu decoctions.** Identified components from **a** to **e** were albiflorin, paeoniflorin, gallic acid, amygdalin and hydroxysafflor yellow A, respectively. Components **f**-**h** were unknown.

Under the present chromatographic and MS conditions, the five significantly changed components were identified or tentatively assigned as albiflorin (**a**), paeoniflorin (**b**), gallic acid (**c**), amygdalin (**d**) and hydroxysafflor yellow A (**e**) by comparing their mass spectrums (Figure 4) with the LC-MS/MS library [12]. To confirm the herbs from which the identified components originated, the ethanol supernate of each constituent herb were also analyzed under the same chromatographic and MS conditions serving as standard references. The ethanol extracts of constituent herbs contained more components than the water extracts both in quantities and in chemical types. There were 6 new components appearing and 23 components dismissing in traditional decoction by consistency comparison. The details of the identified components were summarized in Table 1. The contents of active components albiflorin, paeoniflorin and amygdalin in THSWD were greatly higher than those in THSWDH. The contents of hydroxysafflor yellow A and a unknown component h in THSWD were significantly lower than those in THSWDH. As shown in Table 1, most of the significantly changed components came from BS.

**Table 1:** The significantly changed components identified from Tao-Hong-Si-Wu decoction

	**No.**	**t**_**R**_**(min)**	**[M-H]**^**-**^**/ [M + H]**^**+**^**(*m*/*z*)**	**Molecular formula**	**Assigned identity**	**Source herb**
1	**a**	12.97	479.1533	C_23_H_28_O_11_	albiflorin	BS
2	**b**	13.65	479.1570	C_23_H_28_O_11_	paeoniflorin	BS
3	**c**	1.92	169.0128	C_7_H_6_O_5_	gallic acid	BS
4	**d**	11.17	456.1519	C_20_H_27_NO_11_	amygdalin	TR
5	**e**	11.20	611.1598	C_27_H_32_O_16_	hydroxysafflor yellow A	HH
6	**f**	18.08	207.1012	C_12_H_14_O_3_	unknown	CX, DG
7	**g**	36.97	191.1066	C_12_H_14_O_2_	unknown	CX, DG
8	**h**	3.02	120.0808	C_8_H_9_N	unknown	HH, TR, CX, DG, BS

### Linear regression analysis

For traditional decoction, especially categorized formulae are a complex chemical reaction process, and the active components and their contents may change in this process. All the peaks of mass spectrum (THSWD, DG, CX, BS, SD, TR, HH) were extracted and integration by using QuanLynx™. The original data were imported to software SPSS. Linear regression analysis was made by taking peak areas of Tao-Hong-Si-Wu decoction sample as the dependent variable (y) and peak areas of DG, CX, BS, SD, TR, HH as independent variable (X1, X2, X3, X4, X5, X6). The details of the ANOVA were summarized in Table 2.

**Table 2:** **ANOVA**^**b**^

	**Model**	**Sum of Squares**	**df**	**Mean Square**	**F**	**Sig.**
1	Regression	19182387.435	6	3197064.5275	159.76	7.671E-31^aaaa^
	Residual	1000765.348	50	20011.647		
	Total	20183152.435	56			

a. Predictors: (Constant), HH, TR, BS, CX, SD, DG. b. Dependent Variable: TaoHongSiWu decoction. The linear regression equation was Y = − 31.005 + 2.045X1 + 0.231X2 + 0.995X3 + 0.565X4 + 0.585X5 + 0.136X6. Correlation r = 0.974. Linear regression analysis shows significant difference (*P* < 0.01).

In the above regression equation, the coefficient of X1 to X6 could be regarded as the dosage proportion of DG, CX, BS, SD, TR, HH in THSWD. From the 6 coefficients the DG part was greatly larger than theoretical value 1, SD part was equal to 1, and the 4 other herb parts were less than 1 individually. This showed that the chemical composition contents would be the most close to traditional decoction than any other dosage proportion when the part of DG was set at 2.15 times, SD at 0.99 times, CX at 0.20 times, BS at 0.53 times, TR at 0.60 times, and HH at 0.13 times. It was easy to make out that the order of contributions of 6 herbs in Tao-Hong-Si-Wu decoction should be DG > SD > TR > BS > CX > HH.

The clinical dosage proportionality of Tao-Hong-Si-Wu decoction was 1:1:1:1:1:0.667 (DG : CX : BS : SD : TR : HH), which was obvious different with the optimal proportion from the above regression equation. For dispensing granule decoction, if dosage proportionality still followed the traditional ratio, there were much variation for the chemical composition and the contents of bio-active constituents in comparison with the traditional decoction. Therefore, for dispensing granule decoction, the optimal dosage proportionality of Tao-Hong-Si-Wu decoction should be 2.5:0.2:1:0.5:0.6:0.1 (DG : CX : BS : SD : TR : HH) in order to obtain similar chemical constituent contents with traditional decoction.

## Conclusion

In this paper, chemical consistency between traditional and dispensing granule decoctions of Tao-Hong-Si-Wu decoction was rapidly evaluated by UPLC-QTOF-MS coupled with the MarkerLynx software. The results showed that there was obvious difference in chemical components between the two preparing methods following the same dosage ratio. All the peaks of mass spectrum from Tao-Hong-Si-Wu decoction and each herb were extracted and integration by using QuanLynx™. From linear regression analysis the dosage ratio of individual herbs could be optimized to maintain same component contents with traditional decoction as much as possible. The contribution of each herb in Tao-Hong-Si-Wu decoction, and the optimal compatibility proportion of dispensing granule decoction were derived from the linear regression equation. Furthermore, the optimal dosage proportionality of Tao-Hong-Si-Wu dispensing granule decoction was obtained as 2.5:0.2:1:0.5:0.6:0.1 (DG : CX : BS : SD : TR : HH), which guided better clinic application of Tao-Hong-Si-Wu decoction as dispensing granule decoctions usage, and it also provided some experimental data to reveal the compatibility rule of the relative TCM formulae.

## Abbreviations

TCM: Traditional Chinese medicine; HPLC: High performance liquid chromatography; UPLC-PDA-TOF-MS: Ultra performance liquid chromatography coupled with photo-diode array detector and time-of-flight mass spectrometry; SHXXT: San-Huang-Xie-Xin-Tang; DG: Angelicae Sinensis Radix; CX: Chuanxiong Rhizoma; BS: Paeoniae Radix Alba; SD: Rehmanniae Radix Praeparata; TR: Persicae Semen; HH: Carthami Flos; PDA: Photodiode-array detection; ACN: Acetonitrile; ESI: Electrospray ionization; LC: Liquid chromatography; MS: Mass spectrum; THSWD: Tao-Hong-Si-Wu decoction; THSWDH: Dispensing granule decoctions of Tao-Hong-Si-Wu decoction; OPLS–DA: Orthogonal partial least squared discriminant analysis.

## Competing interests

The authors declare that they have no competing interests.

## Authors’ contributions

ES initiated and all authors designed the study. The extraction and method development were conducted by ES who drafted the manuscript. All authors contributed to data analyses and to finalizing the manuscript. All authors have read and approved the final version.
